# Gluteal muscle activation during weight bearing exercises in a group of healthy subjects

**DOI:** 10.1186/1753-6561-6-S4-P37

**Published:** 2012-07-09

**Authors:** X Huang, HP French

**Affiliations:** 1 School of Physiotherapy, Royal College of Surgeons in Ireland, Ireland

## Introduction

Gluteus Maximus (GMax) and Gluteus Medius (GMed) muscles are commonly weakened in hip osteoarthritis (OA), and functional weight-bearing (WB) exercises are frequently prescribed for gluteal strengthening. Muscle activity as an indicator of muscle recruitment can be measured using electromyography (EMG), with recruitment of >45% maximal voluntary isometric contraction (MVIC) required for strengthening. The aim of this study was to compare gluteal muscle activation during three WB exercises. As no participants with hip OA volunteered over the time-frame of this study, methods and results for the symptom-free group only are presented.

## Methods

Nineteen healthy, symptom-free participants (11 men, 8 women; mean+SD age, 48.68+6.66) were recruited. Surface EMG activity of GMax and GMed was measured bilaterally while participants performed 3 exercises: squat (SQ), right step up (RSU) and right step down (RSD). EMG data were normalized to each participant's MVIC. One way Analysis of variance was used to compare mean EMG activity across the three exercises for left and right GMax and GMed. Significance was set at p<0.05.

## Results

All exercises produced mean EMG amplitudes less than 30% MVIC. RSU had the highest recruitment of R GMed (20.72+7.29 %) and R GMax (13.07+10.73) whilst RSD had higher recruitment for L GMed (16.66+6.74 %). Squat produced the lowest activation for both GMax and GMed. There was no significant difference between GMax activation across the three exercises. Significant differences occurred between step-up and squat for GMed. No significant gender difference existed in magnitude of muscle activation during the three exercises.

## Conclusions

Step-up was the most effective exercise for activating gluteal muscles. Our results suggest that these WB exercises may be beneficial for gluteal muscle endurance, rather than strength, training. These findings will be used to compare against activation levels for people with hip OA.

